# Drug resistance pattern of *Pseudomonas aeruginosa* strains isolated from cystic fibrosis patients at Isfahan AL Zahra hospital, Iran (2009–2010)

**Published:** 2012-06

**Authors:** Fard M Forozsh, G Irajian, Takantape Z Moslehi, H Fazeli, M Salehi, S Rezania

**Affiliations:** 1 School of Medicine, Semnan University of Medical Sciences; 2 Department of Microbiology, School of Medicine, Tehran University of Medical Sciences; 3 Department of Microbiology, School of Medicine, Isfahan University of Medical Sciences

**Keywords:** *Pseudomonas aeruginosa*, Cystic fibrosis, VIM

## Abstract

**Background and Objectives:**

Cystic fibrosis (CF) is an autosomal recessive genetic disease. Infections in these patients are mostly caused by three bacteria: *Staphylococcus aureus*, *Haemophilus influenza* and particularly *Pseudomonas aeruginosa*. Carbapenems including antibiotics are used to combat infections with *Pseudomonas aeruginosa*. In recent years, carbapenems resistant strains of *P. aeruginosa* isolated from clinical specimens are being reported. Decrease in drug penetration and production of metalobeta lactamase (MBLS) have been proposed as mechanisms of resistance.

**Materials and Methods:**

In this descriptive study, the population under investigation was 27 patients suffering from CF in Alzahra hospital of Isfahan. Clinical specimens were taken by deep swabbing from throat and data from every patient was recorded in a questionnaire. The specimens were cultured and isolated organisms were identified as *P. aeruginosa* using standard tests. Kirby-Bauer disk diffusion method was used to determine the bacterial drug resistance pattern. Strains of *P. aeruginosa* were checked for production of MBLS using disk impregnated with IPM-EDTA and PCR targeting of *bla*_VIM_.

**Results:**

Among the 27 patients, 7 (26%) had *P. aeruginosa* infection. In total, 11 *P. aeruginosa* isolates were taken. All isolates were susceptible to imipenem, ticarcillin, ciprofloxacin and piperacillin. The lowest scale of susceptibility belonged to ceftazidime (72.2%) followed by tobramycin (45.4%). None of the strains were positive for the *bla*_VIM_ gene.

**Conclusion:**

Isolates of *P. aeruginosa* from CF patients in Isfahan were susceptible to antibiotics during the study period.

## INTRODUCTION

Cystic fibrosis is an autosomal recessive genetic disease (1). The abnormal characteristic of this disease is the movement of water and ions through the epithelial cells that leads to formation of a dense mucosa and decrease in mucosal clearance in the lungs (2). The disease also causes pancreatic and reproductive organ failure (3, 4). Defect in the *cftr* gene which is located on chromosome number 7 is the main cause of CF (2, 5). In patients suffering from CF, because of the failure in chlorine transition in the upper membrane of respiratory epithelial cells, chlorine is engulfed in these cells and will entail severe absorption of sodium and water through the respiratory duct that will cause the mucosa in the channels to lose moisture and become sticky which finally lead to obstruction in the respiratory channels (1). The existence of dense discharges in these patients will limit the movement of the respiratory cilia. As a result, the air channels will not be refined (6, 7). Bacterial colonization in respiratory duct will lead to the enfeebling of lung function (2). Most infections in these patients are mostly caused by *P. aeruginosa* but also by *Staphylococcus aureus*, and *Haemophilus influenza* (2, 8). The outbreak of *P. aeruginosa* infection among patients with CF varies in the world. The rate of infection with this organism among CF patients was reported as 80% in Belgium and Canada (8, 9). Occurrence of cross infections are common (10). The colonization of this bacterium in the lungs of patient with CF following an initial infection leads to destruction of tissue and decrease in respiratory function (11). Antibiotics are the main ways to control the infection (12, 13). However, *P. aeruginosa* is resistant to most antibiotics and this phenomenon has created problems in treatment of patients (14, 15). The carbapenems including imipenem and meropenem are being used to treat infections caused by *P. aeruginosa* (16). Resistance to carbapenems by *P. aeruginosa* strains has been reported in recent years (17). Decrease in drug penetration and creation of effective betalactamase on carbapenems such as metlo beta lactamase (MBLs) was proposed as reasons for such resistance (17, 18).

Classification of MBLs into three sub groups of B1, B2 and B3 is based on molecular structure. The B1 group includes, GIM, VIM, SPM, and IMP (19, 20). Their producing gene is situated on integrons and can integrate into plasmids or chromosomes. Accordingly, bacteria posessing these genes can transform the genes to other pseudomonas and even other Gram negative rods. So far, no inhibitor has been developed for these enzymes. The recognition of these enzymes and the scale of their outbreak in clinical samples are important to prevent the spread of infection. In this study we investigate the presence of MBLs among *P. aeruginosa* isolated from patients with CF using both phenotypic and PCR assays.

## MATERIALS AND METHODS

In this descriptive study, the population under study consisted of patients suffering from CF (n = 27) in Alzahra hospital of Isfahan. The sputa specimens were taken from these patients using deep swabbing of throat. For every patient a questionnaire (containing age, sex, and history of antibiotic use data) was prepared. The specimens were immediately transferred to blood agar and Mac Conkey agar. The media were incubated for 18 -24 hours at 37°C and then *P. aeruginosa* bacteria were detected through biochemical tests and were kept at -20°C until used (20).

### Antimicrobial susceptibility testing

The isolated organisms were subjected to the Kirby-Bauer disk diffusion method (CLSI) using disks containing imipenem, ceftazidime (CAZ), piperacillin (PIP), ciprofloxacin (CIP), ticarcillin (TIC) and tobramycin. *P. aeruginosa* ATCC 27853 was used as control.

Pure colonies of *P. aeruginosa* were inoculated to tube containing TSB broth (Trypticase Soy Broth) so that the turbidity of the media reached 0.5 MacFarland. Then using sterile swab, this bacterial suspension was cultured in Muller Hinton agar and the disk IMP-EDTA was placed in agar area.

### PCR experiments for detection of MBLs gene

DNA was extracted by boiling method (22). The reaction was prepared in final volume of 25 µl containing 2.5 µl, MgCl, 2 mM, mixture of oligonucleotides (0.2 mM), 0.25 mM of each primers (F- GTTTGGTGCCATATCGCAAC and R-ATTGCGCAGCACCAGGATAG) flanking a 382 bp of *bla*_VIM_ gene, 100ng of template DNA, and 2U of Taq polymerase enzyme.

The thermocycler was programmed as follows: Initial denaturation at 94°C for 5 minutes, and 30 cycles of 94° C for one minute, 56.5° C for 30 seconds, 72°C for 20 seconds with the final extension step at 72° C for 5 minutes.

*P. aeruginosa* containing the *bla*_VIM_ gene (Pasteur Institue of Iran) was used as positive control. The PCR product was electrophoresed on 1.5% agarose gel and following ethidium bromide staining was viewed under UV light.

SPSS 10 software was used for statistical analysis of data.

## RESULTS

**Fig. 1:**
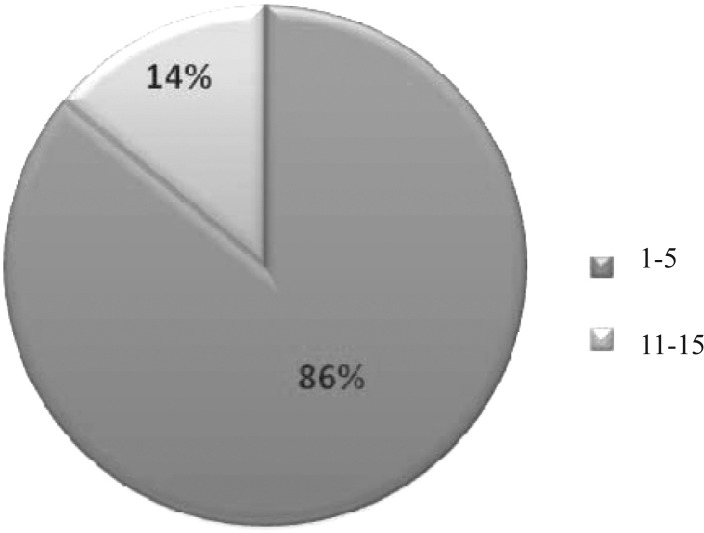
The age groups of cystic fibrosis patients infected with *Pseudomonas aeruginosa*

Of 27 CF patients, 18 (66.6%) were male and 9 (33.4%) were female. Distribution of age groups is shown in Fig. 1. The most prevalent age group belongs to patients 1 to 5 years old and the least were in age group 16 to 25. From the total of 27 patients under study, only 2 (7.4%) had never been hospitalized from birth to the time of sampling and were negative for *P. aeruginosa* in culture. They were female and belonged to the age group 6 to 15. Previous hospitalizations were noticed twice in 11 and three times in 14 of patients. 13 patients underwent respiratory problems at the time of sampling. One patient (4%) had digestive problems and 13 patients (48%) had no notable problem at the time of sampling. From 27 patients, 7 (26%) were infected with *Pseudomonas aeruginosa*. Totally, 11 *P. aeruginosa* isolates were taken (2 patients yielded 3 isolates of *P. aeruginosa*). From 7 patients infected with *P. aeruginosa*, one patient (14.3%) was female and 6 (85.7%) were male. Patients were in age group 1 to 15; however, they were mostly in the 1 to 5 years of age group (86%) and one patient was in age group 6 to 15. No significant relation was found between the frequency of hospitalization and *P. aeruginosa* infection.

**Fig. 2:**
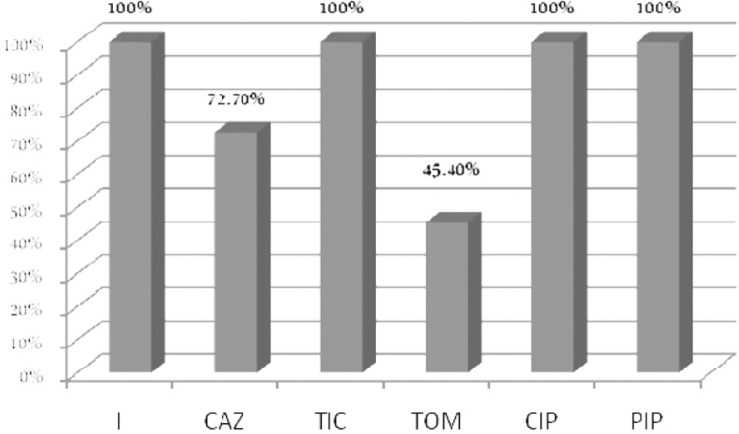
Susceptibility of *P. aeruginosa* isolated from cystic fibrosis patients to antibacterial agents I: imipenem, CAZ: ceftazidime, TIC: Ticarcillin, TOM: Tobramycin, CIP: Ciprofloxacin, PIP: Piperacillin

None of the isolates showed resistance to imipenem. The highest rate of resistance belonged to tobramycin (45.4%). Results of susceptibility testing are shown in Fig. 2. None of the clinical isolates was positive for the *bla*_VIM_ gene.

## DISCUSSION

Bacterial resistance to carbapenems is a clinical concern (20, 23). There is more concern about *P. aeruginosa* which is an opportunistic pathogen in patients with immune deficiency (9, 23). Katarin *et al*. isolated 92 isolates (76.6%) from a total of 120 patients infected with CF in 1996 (24). In Ireland, the prevalence has been reported as 56.2% (25). In Germany, the prevalence of the bacteria in patients having CF has been reported as 50% in 2008 (26). In a 2003 study conducted in Iran, of 64 patients suffering from CF, 21 (32.8%) were infected with this bacterium (27). There are reasons for differences in prevalence of infection with this organism. Hospitalization and duration of infection with *P. aeruginosa* can be a reason since the bacteria can transfer from one infected patient to another (10). Family awareness and educational level of mothers of sick children has a significant role with the spread of infection with *P. aeruginosa* in CF cases.

In this research, all isolates were susceptible to imipenem, ticarcillin, ciprofloxacin and piperacillin. The lowest rate of susceptibility belonged to tobramycin (45.4%) followed by ceftazidime (72.2%). Eftekhar *et al*. did not detect any strain resistance to imipenem in *P. aeruginosa* isolated from patients having CF in Iran in 2003 and reported the rates of susceptibility to ciprofloxacin, ceftazidime, tobramycin, piperacillin and ticarcillin as 7.5, 85.9, 85.7, 81 and 76% respectively (27). It appears that the scale of antibiotic susceptibility of *P. aeruginosa* recovered from people suffering from CF has almost been similar in Tehran and Isfahan. Patients 1-5 years old made the dominant group in both studies. Since the studied group of children had not been bedridden, this may be a reason why multi drug resistant strains of *P. aeruginosa* could rarely be isolated from them. In a study conducted in Portugal, *P. aeruginosa* producing MBLs were reported from a 14 year old patient with CF (28). Isolates of *P. aeruginosa* producing *bla*_VIM_ has already been reported in Iran (Shahcheraghi et al 2010). Because of increase in resistance of *P. aeruginosa* to carbapenems in Iran, it was assumed that we can see the resistance strains of *P. aeruginosa* isolated from patients with CF. However, this hypothesis was not true. It seems that age of patients, hospital stay and contact with CF cases might be risk factors to acquire carbapenems resistant strains of *P. aeruginosa*. All our patients were ambulant and none of them was bedridden at the time of sampling. Most of them (86%) were children from 1 to 5 years old and had less chance of hospitalization and thus the possibility of getting resistance genes in strains isolated from them was far from imagination. It seems that carbapenems remain as effective drug against *P. aeruginosa* isolated from children with CF in Iran.
